# Vertebral Involvement in Pediatric Burkitt Lymphoma: A Case Report

**DOI:** 10.7759/cureus.91924

**Published:** 2025-09-09

**Authors:** Abir Azirar, Ayad Ghanam, Manal Azizi, Imane Kamaoui, Maria Rkain

**Affiliations:** 1 Department of Pediatric Medicine, Centre Hospitalier Universitaire Mohammed VI, Oujda, MAR; 2 Department of Pediatric Medicine, Faculty of Medicine and Pharmacy, Mohammed I University of Oujda, Centre Hospitalier Universitaire Mohammed VI, Oujda, MAR; 3 Department of Radiology, Faculty of Medicine and Pharmacy, Mohammed I University of Oujda, Centre Hospitalier Universitaire Mohammed VI, Oujda, MAR; 4 Department of Pediatrics, Faculty of Medicine and Pharmacy, Mohammed I University of Oujda, Centre Hospitalier Universitaire Mohammed VI, Oujda, MAR; 5 Pediatric Gastroenterology, Centre Hospitalier Universitaire Mohammed VI, Oujda, MAR

**Keywords:** burkitt lymphoma, case report, pediatric lymphoma, spinal cord compression, vertebral involvement

## Abstract

Burkitt lymphoma (BL) is a highly aggressive B-cell non-Hodgkin lymphoma (NHL), and primary spinal involvement as its initial manifestation is extremely rare in children. We report the case of a 10-year-old female presenting with progressive lower limb weakness and back pain. MRI revealed a destructive lesion of the thoracic spine with epidural extension and spinal cord compression. Histopathological examination of a biopsy specimen confirmed the diagnosis of BL. The patient was treated according to the French African Pediatric Oncology Group-Lymphomes Malins B (GFAOP/LMB) protocol, with rapid neurological improvement and complete remission at follow-up. This report highlights the importance of considering BL in the differential diagnosis of pediatric spinal cord compression, as early diagnosis and prompt initiation of appropriate chemotherapy are crucial for achieving favorable outcomes.

## Introduction

Burkitt lymphoma (BL) is a highly aggressive small non-cleaved B-cell malignancy, distinguished by its rapid clinical progression, early hematogenous dissemination, and marked tendency to involve both the bone marrow and the central nervous system (CNS) [1]. The spinal epidural space is a rare initial site of presentation in non-Hodgkin lymphoma (NHL), representing approximately 9% of spinal epidural tumors and only 0.1-3.3% of all lymphomas [2,3]. In such rare presentations, the differential diagnosis of spinal cord compression syndromes should be considered and includes other malignant tumors (such as Ewing sarcoma, neuroblastoma, and metastatic disease), infectious causes (e.g., epidural abscess, tuberculosis), and non-malignant conditions (such as epidural hematoma or transverse myelitis). Distinguishing among these entities requires careful assessment of clinical features, neuroimaging findings, and histopathological confirmation.

According to the WHO classification, BL is categorized into three clinical variants: endemic, sporadic, and immunodeficiency-associated. The endemic form predominantly affects African children, typically between the ages of four and seven years. The sporadic variant is observed worldwide, accounting for 1-2% of adult lymphomas and up to 40% of pediatric lymphomas in the United States and Western Europe. The immunodeficiency-associated form primarily occurs in individuals infected with HIV [4]. CNS involvement is identified in fewer than 15% of sporadic BL cases at diagnosis. It may manifest as meningeal infiltration, cranial nerve involvement, intraparenchymal brain lesions, or the presence of a paraspinal mass [5]. We report the case of a 10-year-old female with developmental delay who presented with spinal cord compression as the initial manifestation of BL, highlighting a rare and severe form of CNS involvement.

## Case presentation

A 10-year-old female patient, with a past medical history of psychomotor developmental delay, presented with a three-week history of progressive lower limb weakness and gait instability. The neurological symptoms had evolved gradually, beginning with mild difficulty walking, followed by increasing stiffness of the legs, frequent falls, and, in the week before admission, marked difficulty standing without support. There was no history of back pain, urinary retention, or sensory changes. On admission to our neurology department, clinical examination revealed a pyramidal syndrome characterized by spasticity and hypertonia predominantly affecting the lower limbs, brisk deep tendon reflexes, and bilateral Babinski signs. No sensory deficits were noted. These findings, in the context of subacute progression, represented neurological red flags for spinal cord compression. On abdominal examination, two firm, palpable masses were identified, along with sub-centimeter lymphadenopathies in peripheral lymph node regions.

**Figure 1:**
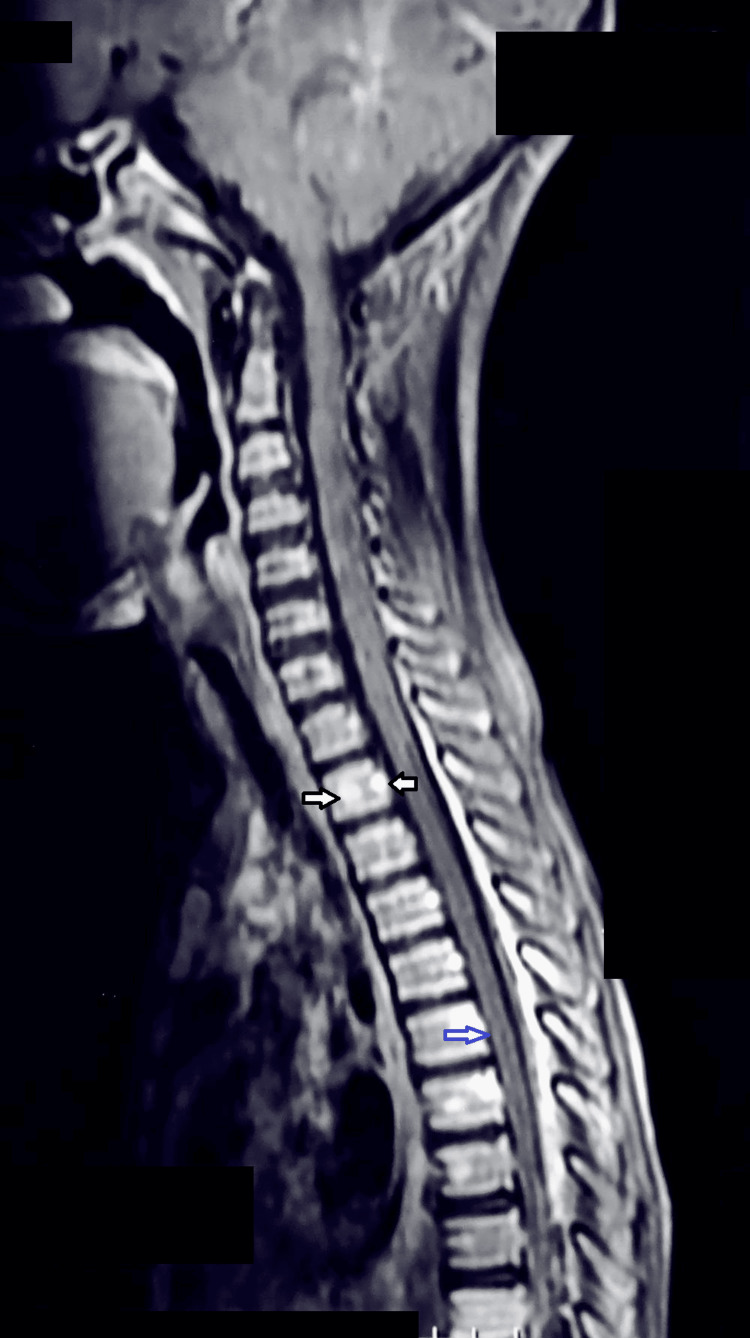
Sagittal T1-weighted MRI of the spine showing vertebral involvement (white arrows) and associated spinal cord compression (blue arrow) MRI: magnetic resonance imaging

To investigate both the neurological symptoms and abdominal masses, a brain and spinal MRI was performed. The MRI revealed multiple vertebral bone lesions extending from the second thoracic vertebra (T2) to the first sacral vertebra (S1), suggestive of lymphomatous infiltration, with associated spinal cord compression. No significant intracranial lesions were detected (Figure 1).

**Figure 2:**
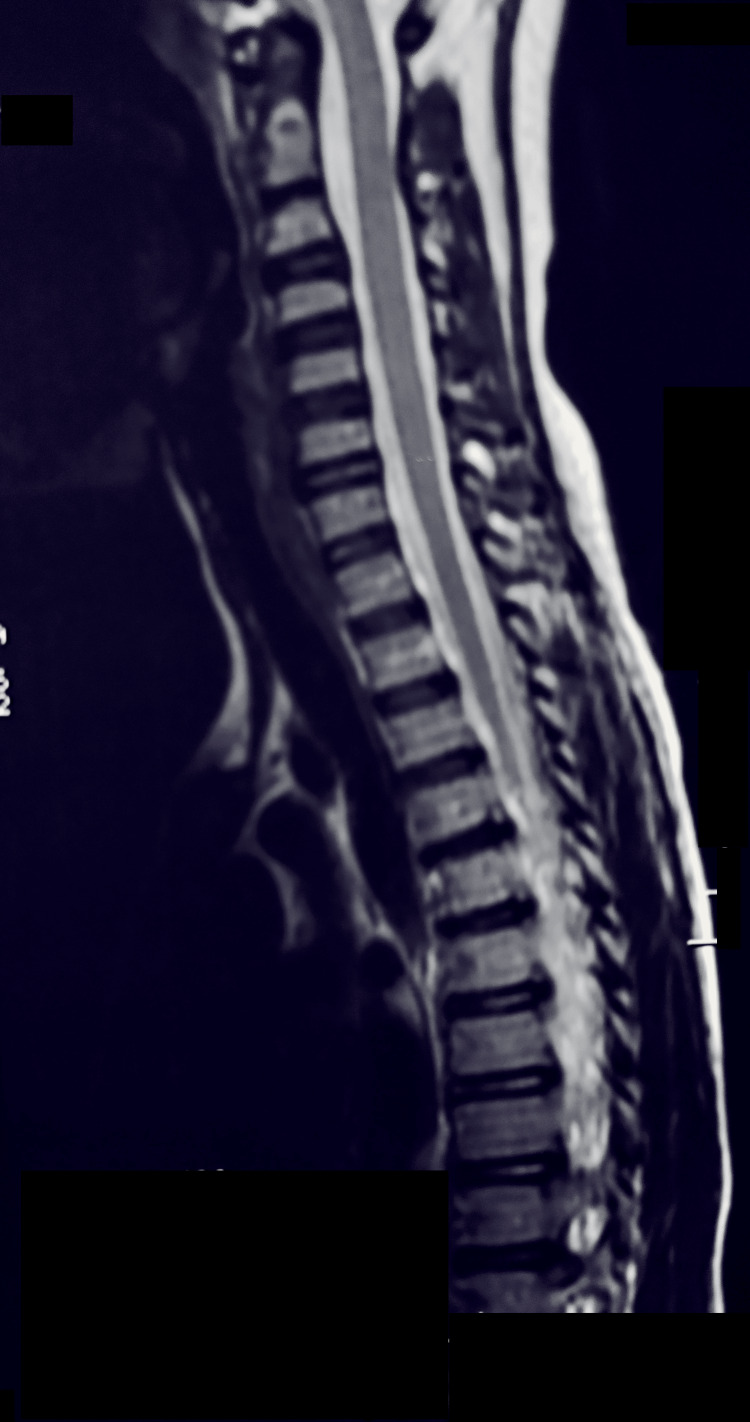
Sagittal MRI of the cervical and upper thoracic spine showing normal vertebral alignment, intact intervertebral discs, and no evidence of spinal cord compression or osseous lesions MRI: magnetic resonance imaging

A fine-needle aspiration biopsy of one abdominal mass confirmed the diagnosis of BL through cytological and immunohistochemical analysis. The patient was immediately started on an intensive multi-agent chemotherapy regimen according to the French African Pediatric Oncology Group-Lymphomes Malins B (GFAOP/LMB) protocol. The clinical course was favorable, with neurological improvement beginning within days of initiating chemotherapy. Over the following weeks, the patient's motor function progressively recovered, gait normalized, and spasticity resolved. A follow-up spinal MRI demonstrated marked regression of the vertebral lesions and complete resolution of spinal cord compression, correlating with the disappearance of the initial neurological deficits (Figure 2).

## Discussion

BL is a highly aggressive B-cell NHL predominantly affecting children and adolescents worldwide. The sporadic form of BL, as seen in our patient, accounts for approximately 30-40% of pediatric NHL in Western countries. It typically manifests with abdominal masses in 60-80% of cases, reflecting the common extranodal involvement of the gastrointestinal tract and associated lymph nodes. CNS involvement is reported in about 15% of cases at diagnosis, usually presenting as leptomeningeal disease or intracranial masses [1,6]. In contrast, vertebral bone involvement with resultant spinal cord compression as the initial presentation remains an exceptionally rare clinical scenario, reported in fewer than 3% of pediatric NHL cases [3,7]. In such rare presentations, the differential diagnosis of spinal cord compression syndromes should be considered. This includes malignant tumors (e.g., Ewing sarcoma, neuroblastoma, metastatic lesions), infectious causes (e.g., epidural abscess, spinal tuberculosis), and non-malignant conditions (e.g., epidural hematoma, transverse myelitis). Differentiation relies on the combination of clinical features, imaging findings, and histopathological confirmation. Comorbidities such as developmental delay, immunodeficiency, or other systemic conditions may further complicate the clinical picture and delay diagnosis [4].

In our patient, the clinical presentation evolved over three weeks, beginning with mild gait disturbance and progressing to significant lower limb weakness, spasticity, and frequent falls. On admission, neurological examination revealed pyramidal signs, including hypertonia predominantly affecting the lower limbs, brisk deep tendon reflexes, and bilateral Babinski signs, representing key neurological red flags for spinal cord compression. Abdominal examination revealed two firm, palpable masses measuring approximately 6 × 5 cm and 5 × 4 cm, along with sub-centimeter peripheral lymphadenopathies. The diagnosis was confirmed by cytopuncture biopsy of an abdominal mass, with immunohistochemistry demonstrating a classical BL immunophenotype (CD10+, CD20+) and a Ki-67 proliferation index near 100%. This profile helps distinguish BL from other aggressive lymphomas and guides treatment decisions [4,5].

This case underscores the importance of considering BL in the differential diagnosis of progressive neurological deficits with spinal lesions in children. Early imaging and recognition of neurological red flags are critical to prevent irreversible deficits. The case also highlights the potential for full neurological recovery with prompt, intensive chemotherapy, even in cases of severe spinal cord compression [7,8,9]. Integrating clinical, radiological, and pathological data strengthens diagnostic accuracy and guides optimal management [4,5].

Treatment was promptly initiated using the GFAOP/LMB intensive chemotherapy protocol, which has demonstrated high efficacy in pediatric BL, including in resource-limited settings [10]. The patient showed rapid neurological improvement, with gradual recovery of motor function, and follow-up MRI confirmed marked regression of vertebral lesions and resolution of spinal cord compression. Similar cases of pediatric BL with spinal involvement are exceptionally rare in the literature. Reports from Morocco and other regions describe isolated epidural or vertebral involvement necessitating urgent intervention [8,9,10]. This report contributes to the limited body of evidence on the topic, highlighting the feasibility of early chemotherapy as a non-surgical approach for recovery and reinforcing its educational value for pediatric clinicians.

## Conclusions

BL with spinal cord compression from vertebral involvement remains an uncommon but critical diagnosis in pediatric patients presenting with progressive neurological symptoms. Early clinical suspicion, thorough imaging evaluation, and timely histopathological confirmation are vital to initiate prompt treatment and prevent irreversible neurological damage. This report illustrates that, despite the severity of the initial presentation, the patient’s rapid neurological improvement reflects the high chemosensitivity of BL, and favorable neurological and radiological outcomes are achievable with early diagnosis and appropriate management. Awareness of this rare presentation can facilitate faster recognition, guide timely initiation of intensive chemotherapy, and ultimately improve prognosis in affected children.
